# Show Me Some ID: A Universal Identification Program for Structural Equation Models

**DOI:** 10.1017/psy.2025.19

**Published:** 2025-04-24

**Authors:** Michael D. Hunter, Robert M. Kirkpatrick, Michael C. Neale

**Affiliations:** 1 Department of Human Development and Family Studies Pennsylvania State University University Park, PA 16802; 2 Virginia Institute for Psychiatric and Behavioral Genetics Virginia Commonwealth University Richmond, VA 23298

**Keywords:** model identification, National Longitudinal Survey of Youth (NLSY), structural equation model (SEM)

## Abstract

With models and research designs ever increasing in complexity, the foundational question of model identification is more important than ever. The determination of whether or not a model can be fit at all or fit to some particular data set is the essence of model identification. In this article, we pull from previously published work on data-independent model identification applicable to a broad set of structural equation models, and extend it further to include extremely flexible exogenous covariate effects and also to include data-dependent empirical model identification. For illustrative purposes, we apply this model identification solution to several small examples for which the answer is already known, including a real data example from the National Longitudinal Survey of Youth; however, the method applies similarly to models that are far from simple to comprehend. The solution is implemented in the open-source OpenMx package in R.

## 1 Introduction

Model identification is a vitally important aspect of all model building. To the extent that vast swaths of research depend on model building, these areas similarly depend on model identification. Informally, a model is identified when the parameters of a model have unique estimates. When a model is not identified, more than one set of parameter values—often infinitely many values—provide identical fit to a set of data.

There are obvious practical problems that arise when a model is not identified, but there are theoretical concerns as well. On the practical side, a model that is not identified might yield different parameter estimates when subjected to repeated model fitting of the same model to the same data. Similarly, the optimization method used for determining parameter estimates might produce extreme and implausible values for some or all of the parameters. Likewise, standard errors that are valuable for statistical inference might be missing, negative, or absurd values. Finally, the software might output the often-dreaded, cryptic “Hessian is non-positive definite” message. For all of the previously-mentioned practical problems, a non-identified model is not the only culprit, but it often stands in line among the usual suspects.

On the theoretical side, the question of model identification determines which models are even possible to fit. Thus, some theoretical questions cannot be answered definitively because the model that answers those questions is not identified. Furthermore, non-unique parameter estimates might not be able to distinguish between competing theoretical explanations of data. The test of a theory might depend on the parameters of a model which in turn depend on a research design that identifies these parameters. Pragmatically, a researcher can plan their data collection design to ensure their theoretically-inspired model is identified. Failure to take model identification into account during the planning phases of research (grant writing, data collection, pre-registration, etc.) can lead to massive wasted resources and slow the progress of scientific knowledge.

Previous work on model identification has allowed researchers across a wide array of social science disciplines to build and fit models that test important research questions. Much of this work began in econometrics with procedures for determining whether systems of linear and nonlinear equations could be solved (Fisher, 1963, 1965; Koopmans, 1949; Wald, 1950) and culminating in a classic book on the topic by Franklin Fisher (1976). Critically, this work was often limited to systems of equations involving strictly observed variables and free parameters, with no latent variables or factors.

Along a separate track, identification procedures for latent variable factor models began with tests of the convergence properties of iterative procedures for estimating these models (Jöreskog, 1967; Lawley & Maxwell, 1963), and further developed into some inspection techniques for finding trade-offs between pairs of parameters (Jöreskog, 1970, p. 247) and the number of restrictions necessary for identification (Jöreskog, 1978, p. 456). Eventually, cross-pollination between economic, sociological, and psychological statistics led to the development of a general model for covariances among multiple variables, including latent variables (Duncan, 1966; Jöreskog, 1970, 1971, 1978; Jöreskog & Goldberger, 1975). These models and their close relatives collectively became known as structural equation models (SEMs).

Today, SEMs are one commonly used tool for the development and testing of theories in social and behavioral sciences. In his landmark SEM textbook, Bollen (1989) presented and developed a number of identification rules for models without latent variables (p. 104), confirmatory factor models (p. 247), and general SEMs (p. 332). However, these rules were far less specific for models with latent variables than those without. In particular, no rule provided in this popular book was both necessary and sufficient for identification for models with latent variables. A well-known necessary-but-not-sufficient identification rule is what Bollen (1989) call the “*t* Rule”: namely, that the number of estimated free parameters in an SEM must be less than the number of unique elements of the covariance matrix. In the parlance of Rodgers (2019), the *t* Rule is a check for positive degrees of freedom, that there is enough statistical capital (degrees of freedom) to “pay for” (estimate) the model. Although advances in SEM identification have occurred (e.g., Davis, 1993; O’Brien, 1994; O’Brien, 1994; Reilly, 1995; Rigdon, 1995) and some books cover this topic extremely well (e.g., Skrondal & Rabe-Hesketh, 2004; Wansbeek & Meijer, 2000) modern SEM books and instruction continue to rely on a series of makeshift, incomplete identification procedures (e.g., Little, 2024; Loehlin, 2004; Maruyama, 1998).

For far too long, identification of SEMs has been plagued by heuristics, half-truths, supposed deep mysteries, and incomplete “rules of thumb.” The basic criterion for identification used in the present article was first established over 70 years ago by Abraham Wald (1950), yet it is not widely known or used for identification of SEMs. The present article provides an analytic solution to data-independent model identification for completely general SEMs, and makes this solution available in the open-source OpenMx (Neale et al., 2016) software. Moreover, we provide a solution for local model identification of SEMs that has clear implications for empirical identification, and apply this solution both to several common longitudinal model structures and to an empirical application on cognitive ability data from the National Longitudinal Survey of Youth (NLSY).

Because a very large class of SEMs make parametric models of the multivariate Gaussian distribution, model identification is actually a long-solved problem, yet the solution is not widely known or easily available in commonly-used statistical software. Although publicizing this solution is not the only contribution of the present work, it may be the most important. The *new* contributions of the present work are threefold. (1) We broaden the SEM identification solution to models with a very general kind of exogenous covariate effect called definition variables. (2) We propose a new method for identification depending on the pattern of observed and missing values in the data. Finally, (3) we provide an open-source software implementation of the above identification methods, including a less-known previously existing method that reports which parameters are not identified, if any.

The structure of this article is as follows. First, we provide a broad way of thinking about SEMs that facilitates later procedures for model identification. Second, we describe the previously published solution to local model identification for parametric models of the multivariate Gaussian distribution. Third, we extend these previously published results to identify models with special exogenous variables in the data—called definition variables—that arbitrarily alter model characteristics. We implement both the previously published solution and its novel extension in the open source OpenMx (Neale et al., 2016) R (R Core Team, 2023) package for extended SEMs as a function called mxCheckIdentification(). Fourth, we use several classic models from longitudinal data analysis to illustrate the model identification solution, emphasizing its strengths and limitations. Fifth and finally, we apply this identification procedure to a model of cognitive development in the NLSY. In this empirical illustration, we show that the standard method of local identification fails to account for problems with empirical identification, but that a further extension of data-independent model identification to some aspects of empirical identification is quite possible.

## 2 A general conception of SEMs

Broadly, an SEM is a parameterized model for a multivariate Gaussian distribution. That is, every SEM implies a means vector 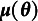 and a covariance matrix 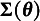 as functions of a vector of free parameters 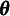. At various times and under varying traditions, different sets of matrices have been used to create 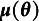 and 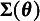. Then the matrices used to create 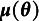 and 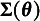 are functions of the free parameters. Each of these sets of matrices can be thought of as a modeling framework for SEM: a way to think about all SEMs.

Examples of important sets of such matrices are the factor model, the linear structural components (LISCOMP; Muthén & Satorra, 1995) model, and the reticular action model (RAM; McArdle & McDonald, 1984), just to name a few.^1^ The factor model has 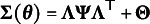 for factor loadings 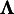, factor covariances 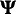, and residual covariances 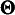. The LISCOMP model has 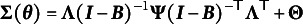 which extends the factor model with the 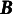 matrix of regression effects between factors. Finally, the RAM has 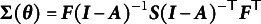
where 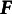 filters latent versus observed variables, 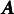 contains all asymmetric relations (i.e., unidirectional regressions) between variables, and 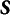 contains all symmetric relations (i.e., bidirectional variances and covariances) between variables. The means implied by the factor model, the LISCOMP model, and RAM follow similar patterns to the implied covariances.

In each of these sets of matrices, the free parameters determine the matrices which in turn determine the expected means and covariances. In the factor model, 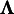, 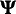, and 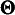 are functions of free parameters which create the model-implied means and covariances. The pattern is similar for the LISCOMP and RAM sets as well. It can be useful to think of a chain of functions that maps free parameters to model-implied means and covariances. In the factor model, 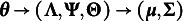. In the LISCOMP model, 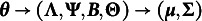. In RAM, 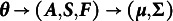.

All of these sets of matrices are sufficiently general to specify any SEM. With regard to the model identification approach we outline, the particular set of matrices is largely irrelevant. The key feature of SEM identification is not that a particular set of matrices is used; it is not that any of these matrices have some set of special characteristics or properties. Rather, the key feature is how the free parameters create the model-implied means and covariances. If this mapping from free parameters to model-implied moments has certain properties, then the SEM is identified.

Consider the mapping from the free parameters to the model-implied means and covariances. Mathematically, we define 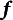 as in Equation (1). 
(1)

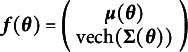

That is, 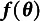 is a function that maps the free parameters of an SEM to the combined vector of the model-implied means and the unique elements of the model-implied covariance matrix (i.e., the half-vectoriztion denoted by 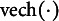 ). The property of the function 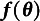 needed for local identification of an SEM is the mapping from the free parameters to the model-implied means and covariances must preserve the full dimension of the free parameters. This property is known as the rank criterion for local identification (Bekker & Wansbeek, 2001; Wald, 1950). Although other ways of determining identification exist (e.g., the existence of the inverse information matrix; Rothenberg, 1971), we focus on the rank criterion for its ease of understanding, ease of computation, and highly useful diagnostics.

## 3 Identification identified

An intuitive understanding of model identification holds that each parameter of a model has a unique, separable effect on the fit of the model, that no parameters can trade-off to create equivalent effects. The official term for these parameter trade-offs is *observational equivalence*, a concept which is used to formally define model identification. A model is identified when there are no observationally equivalent sets of parameter values. Although the purpose of the present work is not a formal presentation of model identification, Appendix A provides these more technical details.

For the present purposes, we can think about the function 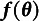 in Equation (1) and how we might investigate its properties. Consider nudging each free parameter and observing how the model-implied means and covariances change in response. Nudge one free parameter and only the variances change; nudge a different free parameter and two variances and several covariances change. We want each free parameter to have its own special effect on the means and covariances that cannot be replicated by other free parameters or combinations of them. This idea of nudging free parameters to find their effects on the model provides the basis for understanding model identification.

Before proceeding, it is important to understand several subtypes of model identification The subtypes of model identification most relevant to the present work are local identification, global identification, and empirical identification. Local and global identification purely deal with the model *per se*, whereas empirical identification depends on features of both the model and the data together. For local identification, there are no observationlly equivalent parameter values only within some local region—technically an open neighborhood—of parameter space, whereas for global identification, there are no observationally equivalent parameter values across the entire parameter space. In empirical identification, the model itself may identified, but it critically depends on certain features of the data which might or might not be present. A model might be locally identified, but not globally identified. However, any globally identified model must necessarily be locally identified. Because local and global identification are features of the *model* and do not depend on the data, we call these kinds of identification data-independent identification and we call empirical identification data-dependent identification. Note that even a globally identified model might be empirically unidentified depending on the data.

Due to theorems that have been proven elsewhere (originally Wald, 1950, p. 244, Theorem 3.3; see Bekker et al., 1994 and Bekker & Wansbeek, 2001 for modern treatments; and see Rigdon, 1997 for a brief review), we know that a model is locally identified at some particular set of values for the free parameters 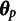 if and only if the matrix of first derivatives of 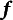 at 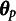 has full column rank. In essence, this full column rank requirement means that any small change in the free parameters has a unique and separable effect on the model-implied means and covariances: that no linear combination of free parameters can trade off to produce the same resulting model-implied means and covariances as another linear combination of free parameters. Nudging each free parameter has a different effect from nudging any other free parameter.

Suppose a model has *p* observed variables and *J* free parameters. Thus, there are 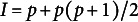 model-implied means and covariances. We will call these means and covariances the summary statistics. Although the model-implied means and covariances are not technically statistics, we use the term “model-implied summary statistics” to evoke their counterparts which are estimated from data, and to not confuse them with the free parameters of a model. Because 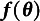 maps *J* dimensions to *I*, the matrix of first derivatives is called a Jacobian and has the general structure shown in Equation (2) 
(2)

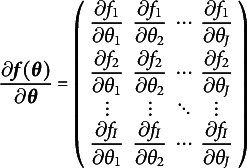

where 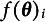 is the *i*th summary statistic, and 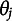 is the *j*th free parameter. This Jacobian directly instantiates the notion of nudging each free parameter and observing what effect is has on the summary statistics. The *j*th column nudges the *j*th free parameter and records its effect on all of the summary statistics, one for each row. The rank of the matrix in Equation (2) then must be equal to the number of free parameters for a just-identified model and greater than the number of free parameters for an identified model. If the derivative values are known, the rank of this Jacobian can be efficiently computed with any rank-revealing 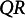 decomposition (e.g., Lay, 2003, pp. 402–426).

The same theorems that derive the conditions for local identification via the rank of the Jacobian show that the null space of the Jacobian yields the set of non-identified free parameters. Thus, the Jacobian not only indicates whether or not a model is locally identified, but also indicates which free parameters are not locally identified. Knowing which free parameters cannot be uniquely determined is often hugely beneficial to researchers when debugging issues with model non-convergence or when building preliminary models for which the identification is not fully understood by the researcher. Again, if the derivative values in Equation (2) are known, then the null space is efficiently computed by any of the many algorithms for the 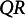 -decomposition.

Analytically computing the derivatives in Equation (2) requires either (a) symbolic matrix calculus on a computer or (b) researcher knowledge of matrix calculus. For a set of simple SEMs, these derivatives are known and can be computed analytically. For example, a simple version of the factor model has a closed form identification reported by Bekker and colleagues (Bekker, 1986; Bekker & ten Berge, 1997). However, the general case of *any* set of matrices combined in an arbitrary way to produce a set of means and covariances is far from solved. An alternative strategy from closed-form analytic solutions is to numerically compute all the derivatives required by Equation (2). Fortunately, the vast majority of situations applied modelers face are very easy and relatively fast to compute numerically (e.g., with Richardson extrapolation; see, Fornberg & Sloan, 1994). Therefore, we need not rely on the specific form of the model or the structure of its free parameters. A custom-built identification method for specialized sets of models might be faster and more efficient for those special cases (cf. Hunter et al., 2021), but the numerical approach outlined here can determine the identification of a much broader class of models.

## 4 Identification generalized

Equations (1) and (2) were shown for the case of all continuous observed variables, a single group, and without constraints, however the same methodology extends to all these cases.^2^ In the case of ordinal variables, Hunter et al. (2023, Appendix B, p. 55) showed that ordinal variables require only a slight alteration to the usual mean and covariances summary statistics by providing full analytic criteria for identifying ordinal variable SEMs. Although a detailed description of ordinal variable identification is beyond the scope of the present work, Hunter et al. (2023, Appendix B, pp. 55–58) provided full mathematical derivations for this situation. We only state their conclusions here. Briefly, underlying each ordinal variable, we assume there is an underlying continuous, latent, Gaussian^3^ variable. These underlying continuous latent variables have no intrinsic scale and thus can be assumed *without loss of generality* to be standard normal (i.e., with means of zero and variances of unity). This assumption does not limit the researcher from choosing any of 13 possible families of scaling for ordinal variables in their models that are not standard normal (see Hunter et al., 2023, p. 57 for details), although some software limits these choices. To identify ordered categorical variables, thresholds that determine the boundaries between ordinal responses must be added to the summary statistics.^4^ The thresholds can all be gathered in a matrix 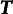 ^5^. Thus, for ordinal variables the appropriate mapping between free parameters and summary statistics is 
(3)

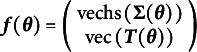

where 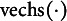 is the strict half-vectorization that omits the diagonal elements of a matrix and 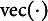 is the full vectorization that concatenates all the elements of a matrix. The combination of some ordinal and some continuous variables (Pritikin et al., 2018) can be handled by appropriately combining Equations (1) and (3) such that continuous variables have means and variances included in the summary statistics but ordinal variables have only covariances and thresholds.

Just as this method of identification applies to ordinal and continuous variables, it also applies to models with constraints and multiple group models. Parameter equality constraints—where one parameter appears in multiple model matrices—directly reduce the dimension of the free parameter vector 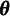 and require no special handling. For all other constraints, let 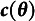 be a vector-valued function of the free parameter vector (cf. Satorra & Bentler, 2001, p. 509). Each element of 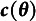 is a univariate, possibly nonlinear constraint function. Essentially, each univariate constraint acts as a new observed statistic. So, the function for the summary statistics and the function for the constraints combine as in Equation (4) (Magnus & Neudecker, 1988, pp. 334–336): 
(4)

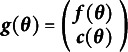

Then the Jacobian is similarly the concatenation of the Jacobian for the summary statistics with respect to the free parameters and the Jacobian of the constraint functions with respect to the free parameters as in Equation (5): 
(5)

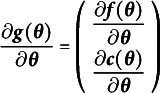

The rank of the matrix in Equation (5) then must be equal to the number of free parameters for a just-identified model and greater than the number of free parameters for an identified model.

A similar concatenation process solves the multiple group SEM problem. If 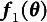 is the mapping from all the free parameters across all groups to the summary statistics for group 1 and 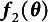 is the mapping from all the free parameters across all groups to the summary statistics of group 2, then the new function 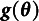 simply combines these as in Equation (6): 
(6)

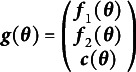

The Jacobian of 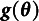 in Equation (6) follows the same pattern as has been shown in Equations 2 and 5. For completeness, the Jacobian is shown in Equation (7). Of course, this two-group situation extends to arbitrarily many groups. 
(7)

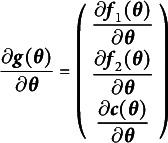



The mathematical and theoretical procedures for SEM identification which we reviewed above have been known for quite some time. With modern computing, the identification of even large SEMs of many variables and many parameters presents no computational difficulty. No doubt, the lack of software tools for identification of these models has impeded progress in the social and behavioral sciences. The OpenMx (Neale et al., 2016) software has included model identification procedures for single and multiple-group SEMs in its mxCheckIdentification() function since version 2.2.2 in 2015, with support for constraints added in version 2.13.2 in 2019. We next consider a novel extension of model identification newly added to OpenMx in version 2.21.12 in 2024.

## 5 Definition variables

The material presented so far is not novel. Although not widely known, the rank of the Jacobian criterion for local model identification has been known in the mixture modeling and SEM contexts for decades (Bekker et al., 1994; Bollen & Bauldry, 2010; Goodman, 1974). A novel contribution of the present work is extending this criterion to the case of so-called definition variables, a special kind of exogenous covariate that can influence the means and/or the covariances of an SEM in quite general ways.

A definition variable is a special kind of exogenous variable that is allowed to modify any part of a model. The term “definition variable” comes from their original software implementation in classic Mx (Neale, 1995) which used #define commands to define the *dimensions* of model matrices, and a Definition_variables command to define some or all of the *values* within matrices in its syntax. Thus, definition variables were variables that defined the model itself, rather than variables that were modeled. In their simplest form, a definition variable replaces a free parameter as an element of a matrix that leads via some algebraic combination to the model-implied means and covariances. For example, instead of a free parameter in the factor loadings matrix of a factor model, an element of the data could replace that factor loading and vary for every row of data in whatever way the data vary. Precisely this substitution allows for individually-varying times of measurement in latent growth curve models (Mehta & West, 2000). Thus, definition variables allow the data to modify the model, potentially for every row of data.

The notion of a definition variable in SEMs dates back to the mid-1990s or earlier when they appeared in the Mx statistical software (Neale, 1995). At the time, the primary application of definition variables was facilitating certain kinds of models in behavior genetics that assess gene-by-environment interaction (e.g., Martin et al., 1987) and sex-limitation (Neale & Cardon, 1992; Neale & Maes, 2004, pp. 211–229). Since that time, definition variables have been applied to examine multivariate gene-by-environment interactions (Neale et al., 2006), higher-order gene-by-environment interactions (Purcell, 2002), person-specific times of measurement in latent growth curves (Mehta & West, 2000), and moderating dynamics in multivariate, latent time series models called state space models (Adolf et al., 2017; Hunter, 2018).

Limited versions of definition variables allow several SEM software programs to include individually-varying times at which measurement occurred in latent growth curve models. These individually-varying times allow SEMs to replicate design matrices for the fixed effects and random effects in linear mixed effects models (Laird & Ware, 1982). Similarly, many software programs for SEM allow for exogenous covariates to linearly influence the means while having no effect on the covariances (e.g., Muthén, 1983, Equations (6)–(13), pp. 45–46). For example, part of the LISCOMP model is often stated as 
(8)

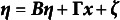

where 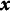 is a vector of exogenous, fixed covariates, not modeled random variables. In Equation (8), 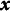 acts as a limited version of a vector of definition variables. Another limited version of definition variables is used in “moderated nonlinear factor analysis” (Bauer, 2017; Bauer & Hussong, 2009; Curran et al., 2014), which allows factor loadings to vary as linear functions of definition variables.

The most general version of definition variables allows *any* matrix in an SEM to vary as any function of both free parameters and definition variables. Replicating the classic Mx software (Neale, 1995), the OpenMx software (Boker et al., 2011; Neale et al., 2016) allows this behavior and therefore identification of these models is also a matter of concern. Previous research has not solved the problem of model identification when there are definition variables, nor—to our knowledge—ever even attempted a systematic approach to its solution.

## 6 Identification with definition variables

The way that SEMs with definition variables are identified relates to the conceptual origins of definition variables themselves. Note that in the classic Mx software definition variables arose for two broad kinds of purposes. First, definition variables allowed for a kind of “multilevel” model (Neale, 1995, p. 20; Neale et al., 1999, p. 46). That is, they allowed the parameters of the model to differ for each row of data, thereby creating row-specific effects akin to random effects in a multilevel model. Importantly, these row-specific effects lacked the distributional assumptions and corresponding computational efficiency of true multilevel models, yet they aimed at a similar purpose of accounting for heterogeneity and dependence across units of analysis.^6^ Second, definition variables allowed for programmatically creating models with a potentially vast number of groups: “effectively as many groups as there are cases in the data file” (Neale et al., 1999, p. 46). That is, each combination of definition variable values could be equivalently treated as a separate group in a multigroup SEM. Identification of SEMs with definition variables proceeds from this multigroup perspective.

To identify an SEM with definition variables, start with the assumption that rows of data are independent. Then definition variables give different summary statistics for each row of data. Rather, definition variables give different summary statistics for each unique combination of definition variable values. The appropriate Jacobian of the mapping between the free parameters and the summary statistics is then extended for each unique combination of definition variable values. An SEM with definition variables is locally identified when the extended Jacobian has full column rank. In essence, an SEM with definition variables is identified by turning each combination of definition variable values into a group, and then identifying the multiple group SEM. Although we present no formal proof of this identification method, the logic is relatively straightforward. Appendix B explains this method further and provides a detailed example that applies this method to the identification of ordinary least squares regression when specified in the conventional way and when specified as an SEM with definition variables.

As a simple initial case, consider a single-group SEM with one definition variable 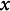 that takes on two distinct values: 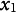 and 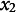 . For example, consider making a model where the means and covariances were allowed to differ by binary sex. Such a model could be parameterized as a multiple group model or equivalently as a model that incorporated sex as a definition variable that influenced the means and covariances. Equation (9) shows the appropriate mapping from the free parameters and definition variables to the summary statistics for a single definition variable with two distinct values. 
(9)

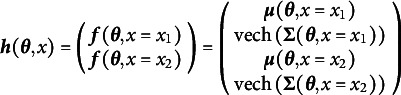

Note the similarity between Equation (9) and its multiple-group and single-group analogs in Equations (6) and (1), respectively. Now the means, variances, and covariances are functions of the free parameters and of the definition variables. The way that free parameters map onto the model-implied summary statistics is generally defined by the researcher-specified model and the modeling framework (e.g., LISCOMP, RAM, COSAN, etc.); whereas the way that definition variables alter the means, variances, and covariances is entirely up to the researcher.

Regardless of the way a researcher decides to let definition variables alter the summary statistics, in Equation (9) the two unique values of the definition variable effectively create two groups of summary statistics. Because the definition variable has only two unique values, we only evaluate the mapping from the free parameters to the summary statistics 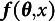 at these two values. The function 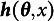 maps the free parameters and definition variables to as many versions of the summary statistics as the definition variables require. In this case, 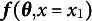 contains the means, variances, and covariances for the model at the free parameter value 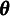 and the definition variable value 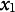 ; 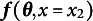 contains the means, variances, and covariances for the model at the free parameter value 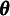 and the definition variable value 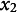 .

Model identification for definition variables reduces exactly to model identification for multiple groups where each unique combination of definition variable values forms a group. For an appropriately defined model the function 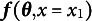 in Equation (9) is exactly equal to the corresponding function 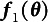 in Equation (6). The summary statistics evaluated at the first definition variable value are equivalent to the summary statistics for a virtual group created for this definition variable value. Definition variables turn single-group models into multiple group models which can then be identified accordingly.

The model considered in Equation (9) allows for different free parameters across binary sex and thus depends on the definition variable taking on distinct values for its identification. As will be seen in the illustrative examples, some models depend on the definition variables taking different values for identification, whereas other models only depend on a single set of definition variable values—even though the definition variables may take on many more values. The models that depend on distinct definition variable values for identification are not locally identified for *any* single value for the definition variables; however, the same models are identified when accounting for distinct definition variable values.

The case of SEMs with multiple groups, constraints, and a single definition variable with two unique values extends similarly. 
(10)

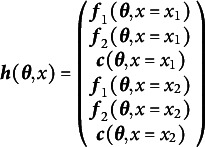

In Equation (10), the function 
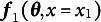
 returns the summary statistics for group 1 when evaluating the definition variable 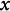 at the specific value 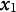 . Accordingly, 
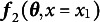
 returns the summary statistics for group 2 with the definition variable value of 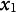 . The other components of Equation (10) follow similarly.

Finally, the most general case of an SEM with multiple groups, constraints, and a vector of multiple definition variables 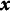 with as many unique combinations as there are rows of data, *N*, is shown in Equation (11). 
(11)

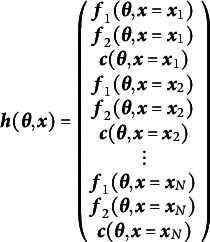

The notation 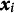 indicates the vector of all the definition variable values at row *i*.

Equation (11) gives the appropriate mapping from the free parameters and definition variables to the summary statistics. The identification of the corresponding SEM is given by the rank of the Jacobian in Equation (12). 
(12)

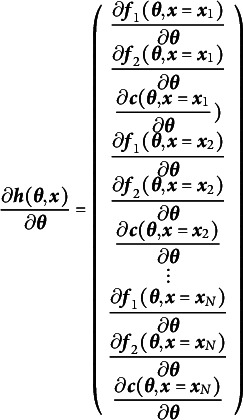



Although the Jacobian in Equation (12) contains a potentially large number of rows, the algorithmic complexity of the computation is quite small. The procedure merely replicates Equation (7) for each unique combination of definition variable values. In practice, even when there are a large number of definition variables with many unique combinations, many models are identified using only one or two unique combinations of definition variable values. One reasonable strategy for determining model identification is to iteratively continue evaluating model identification for each new combination of definition variable values, and to stop adding new combinations once the model is identified. Note that once the Jacobian has rank equal to the number of columns and greater than or equal to the number of summary statistics, the model is identified. Although some further information might be gained by extending the Jacobian to all unique sets of definition variable values, the rank of the Jacobian will never decrease based on this extension. So, no further information about model identification is gained after the model is minimally identified.

An alternative—and more heuristic—approach for computing model identification with definition variables would be to evaluate the Jacobian with two unique definition variable values, and then let the researcher determine if further evaluations are worthwhile. Of course, the maximalist approach of evaluating the Jacobian at every unique combination of definition variable values remains a viable—if cumbersome—option as well.

All the definition variable identification methods rely on the actually observed values of the definition variables, making them dependent on the definition variable data but not the modeled variable data. This form of data-dependence can lead to issues of empirical identification. For example, a model with definition variables might be identified for a particular combination of definition variable values but not for those that are actually observed. These issues of empirical identification are considered next.

## 7 Empirical identification

Up to this point, we have primarily been concerned with model identification as a property of the model itself. The addition of definition variables only slightly modifies the perspective that a model is or is not identified independent of the data used to fit the model. However, with empirical identification we are primarily concerned with data-dependent identification. For example, the identification of a model might depend on the covariance between two particular variables. The model itself might be identified, but if that covariance never occurs in the data (e.g., if only one member of that pair of variables is ever observed), then that model is empirically unidentified for those data even though the model is locally identified in principle.

Unlike local and global identification, “empirical identification” has a relatively ambiguous meaning. Many articles and books do not even mention empirical identification (e.g., Bekker, 1986; Bekker & ten Berge, 1997; Bekker & Wansbeek, 2001; Bekker et al., 1994; Goodman, 1974; Little, 2024; Magnus & Neudecker, 1988; McDonald & Krane, 1977, 1979; McDonald, 1982; Wald, 1950; Wansbeek & Meijer, 2000). Other works use empirical identification to mean local identification at the parameter estimates (e.g., Skrondal & Rabe-Hesketh, 2004), or local identification in general (e.g., Loehlin, 2004, p. 74). Still others refer to local identification as an empirical test or empirical check of identification, which can sometimes be termed empirical identification (Bentler & Weeks, 1980; Bollen, 1989; Bollen & Bauldry, 2010). Finally, some authors use empirical identification to mean a variety of data-dependent issues that may arise in model estimation (Rindskopf, 1984).

For the present work, we define empirical identification as identification over the observed data, rather than over all theoretically possible data. A formal definition is provided in Appendix A. This definition includes local identification at the parameter estimates as a special case, namely the special case of being locally identified for the particular data that yields a particular vector of parameter estimates. This definition also includes a variety of other data-dependent situations that can cause difficulties with model estimation (e.g., multicolinearity), but we focus on the special case of missing data.

We propose a novel method of investigating empirical identification that naturally augments the theoretically strong foundation of local model identification previously discussed. In local model identification we obtain a Jacobian that shows how each free parameter influences the model-implied means and covariances. So, we can immediately see from inspecting this Jacobian that some free parameters have no influence whatsoever on some of the summary statistics. This inspection of the Jacobian for zero entries yields some intuition on which summary statistics are necessary for identification. Automatically constructing a list of such dependencies is an easy task for modern computers.

Beyond intuition, we can use changes in the rank of the Jacobian dependent on removing summary statistics to quantitatively assess the dependence of each free parameter on each summary statistic. By dropping a row of the Jacobian and re-evaluating its rank, we can show how critically the rank depends on each summary statistic. If the rank of the Jacobian changes when a summary statistic is dropped, then that summary statistic was critical for identifying at least one free parameter. In fact, the change in the rank of the Jacobian corresponds to the number of newly unidentified parameters. Moreover, the null space of the Jacobian shows *which* free parameters are no longer identified. Thus, by examining which free parameters become unidentified in response to dropping a summary statistic, we can determine which summary statistics are essential for identification of each free parameter.

With a correspondence between the summary statistics and the free parameters, we can then compare each of the model-implied summary statistics to the observed frequency of non-missing values in the data. A extremely simple case of empirical non-identification would be a model that includes a mean for a variable that is actually all missing. The model might be locally identified, but the mean of that all-missing variable is not empirically identified. This empirical non-identification would be easily detected using this approach. A slightly more complicated case of empirical non-identification would be a model that depends on a particular covariance between two variables, but those two variables are never actually observed together. Again, this empirical non-identification is readily captured by the approach we propose.

Empirical identification is a complicated phenomena. We do not suggest that all possible cases of empirical identification are solved by this approach. However, the approach capitalizes on the strong mathematical foundation of local identification, and certainly captures several common situations where empirical identification causes problems.

## 8 Illustrations of local identification

Although the theoretical and mathematical formalism behind model identification can be daunting, we provide software tools that ease researcher burden when considering whether any particular model is identified. Concrete illustrations of these tools help show both their strengths and shortcomings. We use small, synthetic examples to demonstrate model identification for (1) factor models, (2) latent growth curve models, and (3) variance component models that are commonly used in behavior genetics. Code for all of these example is available online at https://osf.io/zgj82/.

### 8.1 Factor models

Consider a one-factor model with three indicators. There are two common strategies employed for identifying this model. One strategy identifies the latent variable by fixing one factor loading to unity and the factor mean to zero. Another strategy identifies the latent variable by fixing the factor variance to unity and the factor mean to zero. Both methods make the model identified; however, the first strategy makes a factor model that is globally identified, whereas the second strategy makes a model that is only locally identified.

One way to understand this apparent contradiction is to realize that setting the mean and variance of the factor does not fully specify the scale of the latent variable; it leaves the sign of the latent variable ambiguous. The factor can be multiplied by negative one and maintain all the same properties. If the zero mean and unit variance identification strategy is used for any factor model with any number of factors, each factor could be reversed in direction by multiplying all the factor loadings by minus one. If identifying a factor by fixing a loading to one, it is no longer possible to reverse the direction of all the loadings and thus the factor with it. A demonstration script that computes the full Jacobian and shows the identified and non-identified models is available online at https://osf.io/6ezq5.

The full Jacobian of this factor model at a chosen set of parameter values is shown below 
(13)

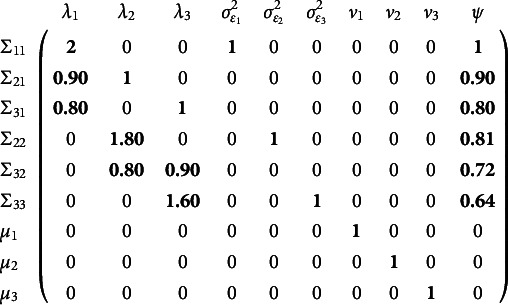

where the factor loadings are 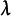 s, the residual variances are 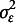 s, the item intercepts are 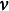 s, and the factor variance is 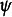 . The Jacobian is shown at the free parameter values of 1, .9, and .8 for the loadings, 1 for all the residual variances, 0 for the intercepts, and 1 for the factor variance. The nonzero elements of the Jacobian are in bold. Critically, some of the numbers in the Jacobian change depending on the chosen free parameter values, but others do not. The equation for the variance of the first observed variable is 
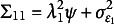
 . The analytic nonzero partial derivatives of this equation are 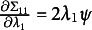 , 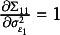 , and 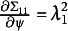 . Evaluating these partial derivatives at the chosen parameter values yields the first row of Equation (13). A similar process applied to all the rows in Equation (13) yields the numbers shown. Note that the columns of Equation (13) associated with the residual variances and the intercepts never depend on the free parameter values chosen.

Shifting from analytic expressions to particular numeric values is a key step in the *local* identification of SEMs. Because the Jacobian is evaluated at a particular set of parameter values, its rank can vary for differing free parameter values. Consequently, an SEM can be locally identified at some parameter values, but not for others. Divergent local identification across parameter values makes the careful choice of those parameter values critical. For a broad class of models and model parameters, evaluating local identification at zero and unity is frequently misleading for this reason.

The rank of this Jacobian in Equation (13) is nine, but there are 10 columns: one column corresponding to each free parameter. So, this model is not locally identified. Simple parameter counting rules would yield the same conclusion that this model is not identified. However, examination of the null space of this Jacobian reveals *which* parameters are not identified and how an identified solution could be obtained. The null space shows that 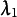 , 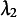 , 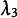 , and 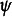 are not simultaneously identified. By inspection, one can see that a linear combination of the 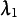 , 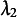 , and 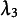 columns can be made to equal the 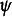 column: suggesting that either fixing one factor loading to a constant value or fixing the factor variance to a constant value would identify the model. The identification that fixes the factor variance drops the last column. The identification that fixes the first factor loading drops the first column. Both of these strategies leave the rank of the Jacobian unchanged at nine, but a rank nine Jacobian with nine observed statistics means the model is now identified.

Finally, when evaluated at factor loadings of zero, the first three columns become all zeros and the factor variance column also becomes all zeros: zero factor loadings mean that changing the factor variance has no effect on the model-implied variances or covariances. Thus, at factor loadings of zero, the rank of the Jacobian reduces from nine to six; only the residual variances and intercepts remain identified. Crucially, the structure of the model was not altered by examining the Jacobian at different values, but the rank of the Jacobian changed from nine to six merely by evaluation at a different point in parameter space. The possibility of creating divergent identification results depending on the values of the free parameters is a persistent limitation of local model identification. One strategy to resolve the problem of locally unidentified models that are identified at other parameter values is to evaluate local identification at several possible parameter values, perhaps randomly generated parameter values. Such a strategy moves parameters off locations in parameter space that are unidentified under the assumption that a model is identified for large proportions of parameter space, but perhaps not for a small number of specific values or combinations of values.

### 8.2 Growth models

Consider a latent growth curve model with three time points. When all people are observed at the same time points, this model is equivalent to a factor model with fixed loadings. It is well-established that the linear latent growth curve model is identified for three time points, but that a quadratic latent growth curve model is not identified. Again, a full demonstration script is available online at https://osf.io/6ezq5.

The full Jacobian for the quadratic latent growth curve models is shown below 
(14)

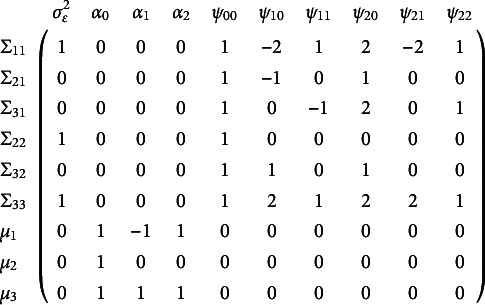

where 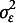 is the residual variance, 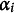 is the mean for the growth factor of polynomial order *i*, and 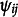 is the covariance between growth factors *i* and *j*. Because the linear growth curve model is a special case of the quadratic growth curve model, the Jacobian for the linear case can be obtained from the quadratic case. The linear growth curve Jacobian is obtained by dropping the columns associated with means, variances, and covariances of the quadratic growth factor: 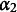 , 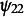 , 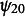 , and 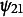 .

Note that simple parameter counting suggests that the quadratic growth curve model is not identified with nine summary statistics and ten free parameters. The Jacobian method finds that this model is not identified, being rank nine, but usefully finds that the following parameters span the null space causing the non-identification: 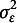 , 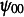 , 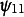 , 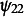 , and 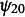 . Inspection of these columns in the Jacobian suggests that one constraint can make them all linearly independent. Constraining the covariance between the intercept factor and the quadratic factor to zero identifies the model by removing one of the ten columns but leaving the rank unchanged at nine. An alternative identification strategy for the quadratic growth curve model with three time points uses definition variables. When this model uses definition variables and the times at which observations occur differ across people, the three-time-point quadratic growth curve model is identified without the need of further constraints.

### 8.3 Variance component models in behavior genetics

A common model in behavior genetics examines a single phenotype (i.e., outcome variable) measured on numerous twin pairs. The twins are either monozygotic (i.e., “identical”) or dizygotic (i.e., “fraternal”). In the most common design, both members of a twin pair were raised together in the same household. This design allows—under certain assumptions (see, e.g., Neale & Maes, 2004)—the decomposition of the means, variances, and covariances into factors that are driven by additive genetic similarity (A), common environmental similarity (C), and unique environmental similarity (E). Hence the ACE acronym is often used to describe this model. The simplest version of this model implies bivariate (one variable for each member of the twin pair) means and covariances as functions of the free parameter vector 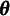 as shown in Equation (15) 
(15)

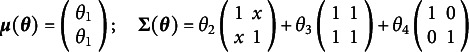

where *x* is a definition variable that is .5 for all dizygotic twin pairs and 1.0 for all monozygotic twin pairs. The 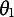 parameter constrains the phenotypic mean to be equal across members of a twin pair; 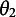 is the variance associated with additive genetics; 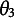 is the variance associated with common environments; and 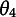 is the variance associated with unique environments. Using analytic methods derived by Hunter et al. (2021), one can show that this model is not locally identified for any single value of the definition variable *x*, but the model is identified when transformed into a two-group model without definition variables. The methods developed in the present work similarly show that the one-group model with definition variables is identified.

The instance of Equation (7) for this model shows that it is not identified for any single value of the definition variable, *x* at row one notated by 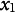 regardless of the free parameter values chosen (see Hunter et al., 2021 for the derivation of this expression and how it is invariant to the chosen free parameter values). 
(16)

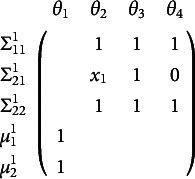

The blanks in Equation (16) are zeros that merely show the sparse blockwise structure and are intended to increase readability. Again, parameter counting suggests this model might be identified, having four parameters and five summary statistics. However, the Jacobian has rank three, not four. The structure of the above Jacobian lets the mean parameter 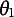 be identified, but not all of the variance parameters: the columns for 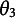 and 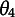 can combine to equal the column for 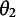 . Further extending the Jacobian as in Equation (12) identifies the model as shown in the Jacobian below. 
(17)

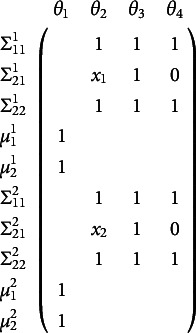

The superscript indicates the newly created group corresponding to distinct values of the definition variable at rows one and two. With this extension, the model is identified because any linear combination that equals the 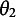 column for the first group does not equal the 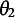 column for the second group. So, the rank is now four with four free parameters, making the model identified. Because the Jacobian in Equation (17) does not depend on the actual free parameter values and only requires that 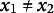 , this model is also globally identified.

## 9 Empirical identification in the NLSY

To demonstrate the proposed method for assessing empirical identification, we apply the method to data from the NLSY. The NLSY is a United States national household probability sample with data initially collected in 1979. We analyze cognitive longitudinal data collected on the children of the females from the original sample ( 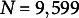 ). The NLSY is rich in numerous assessments, but for the purposes of illustration we examine four variables: reading comprehension, reading recognition, digit span, and mathematical ability. These measures were collected at several time points between ages 3 and 17, but we focus on ages 10, 11, 12, and 13.

### 9.1 Factor model

For simplicity, consider a factor model with one factor at each age. The age 10 factor has four indicators: the scores of the four cognitive tests all at age 10. The remaining three factors are constructed similarly. As has been well-established and can be verified, this model is locally identified by either (1) fixing one factor loading for each factor along with the factor mean or (2) fixing the factor variance along with the factor mean. All the remaining factor loadings, residual variances, item intercepts, and factor covariances can be freely estimated. These free parameters total 54 in number and the rank of the corresponding Jacobian is 54 when evaluated at almost any specific free parameter values, indicating the model is locally identified. However, there is a pattern in the data collection design that means this model is actually not empirically identified.

**Figure 1:**
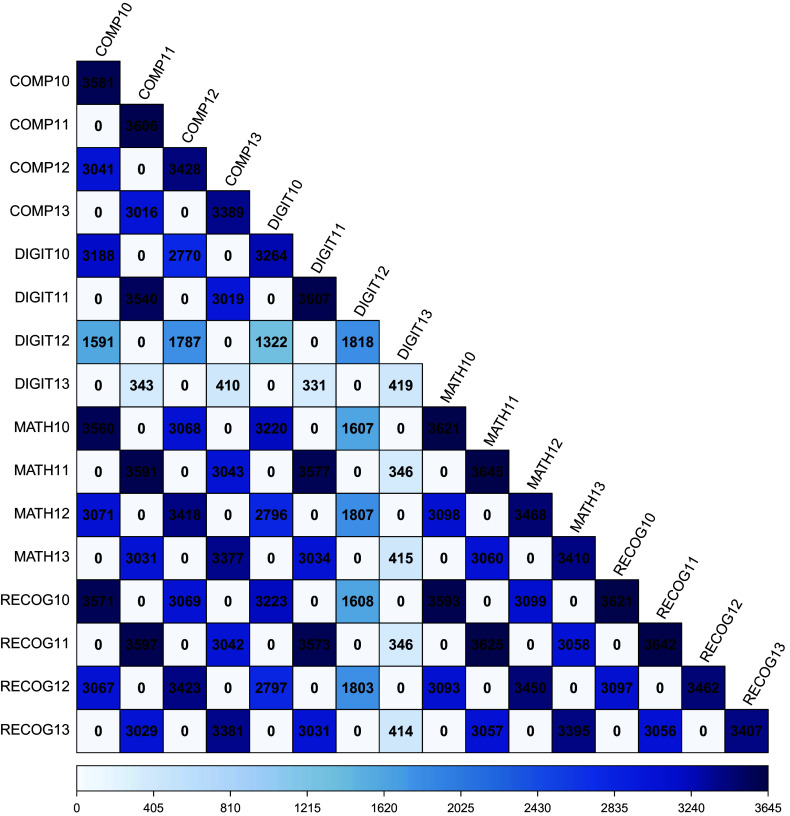
Frequency of non-missing observations in the National Longitudinal Survey of Youth 1979 children sample for several cognitive measures at ages 10, 11, 12, and 13. *Note*: COMP = reading comprehension, DIGIT = digit span, MATH = mathematical ability, RECOG = reading recognition. The suffix for each variable is the age at which assessment occurred. Frequency of non-missing observations is shown both numerically and using shading.

Figure 1 shows the frequency of bivariate non-missing data for each pair of observed variables. As shown, there are a large number of zero frequencies. No individual in the data was observed at age 10 *and* age 11. Observed data only occur for even pairs of ages (10, 12) or odd pairs of ages (11, 13). This pattern of missingness is due to the biennial data collection schedule of the NLSY for these individuals.

We know from first principles that a covariance cannot be estimated when there are no observations. Consequently, we know that we cannot estimate the covariance between any variable at age 10 and any variable at age 11. The situation is parallel for age 10 and 13, age 11 and 12, and age 12 and 13. So, the covariance between the factors defined at these ages also must not be empirically identified. Using the method outlined previously that filters out rows of the Jacobian which have zero frequency, we can create a new Jacobian. This Jacobian still has 54 columns, but after filtering the zero frequency summary statistics has only 88 rows instead of 152 rows. If we relied purely on the count of the observed statistics and the free parameters, we would still say this model is identified. However, computing the rank of the filtered Jacobian yields 50 instead of 54. So, the model is in fact not locally identified due to empirical missing data patterns. Furthermore, the method simultaneously determines which of the free parameters are not identified and states that only the appropriate factor covariances are not identified. Because the factor variances are identified, but not their covariances, one could say that the factor correlations are not identified; however, the model parameters that are not identified are factor covariance parameters. Although we do not present the full 152 by 54 Jacobian here, we do provide demonstration code online at https://osf.io/zmtwu that runs the full analysis.

### 9.2 Growth model

Consider a quadratic latent growth curve model of digit span in the NLSY Children data. Although digit span is measured at four time points (ages 10, 11, 12, and 13), the pattern of biennial data collection throughout the NLSY holds: no child is measured at ages 10 and 11, 10 and 13, 11 and 12, and so on. Moreover, the sample size at age 13 is between 11% and 23% of that at the other ages. A quadratic latent growth curve model is locally identified for a large portion of the parameter space when there are four time points. However, we want to know in what way this identification relies on the structure of the collected data.

We can apply the same filtering technique to the Jacobian that was used in the previous example, and drop the rows associated with the missing covariances. Although the initial rank of the Jacobian was ten for the ten free parameters (one residual variance, three factor means, three factor variances, and three factor covariances), the rank of this filtered Jacobian is only eight. The null space of this filtered Jacobian finds that the residual variance, the factor variances, and the factor covariance between the intercept and quadratic term are not simultaneously identified.

This situation almost exactly mirrors that of the local identification growth model with three time points considered earlier. The strategy employed there was to drop the covariance between intercept and quadratic factors. Dropping this covariance here leaves the rank unchanged at eight, but there are still nine free parameters so the model is still unidentified. In addition to this covariance, one could drop the residual variance, the intercept variance, or the linear slope variance and the resulting model would be identified (rank eight on eight free parameters). But dropping the quadratic variance further reduces the rank to seven on eight free parameters. So, dropping the quadratic term variance is not a suitable identification strategy.

The empirical identification finding for growth models is far less intuitive than that for a simple factor model. Even though there are four time points of data on a single observed variable and the quadratic growth curve model is identified in principle, it is not empirically identified. The quadratic growth curve model critically depends on covariances in the data that are missing by design.

In addition to the simple filtering technique based on zero frequencies of non-missing values, a researcher might want to adjust this threshold to some other value based on a desired minimal sample size for suitable estimation precision. Inspection of Figure 1 shows that there are relatively few observations for digit span at age 13, and even fewer for the covariance between digit span at ages 11 and 13. Dropping the rows of the Jacobian corresponding to the digit span variance at age 13 and its covariance with age 11 further reduces the rank of the filtered Jacobian from eight to seven. The unidentified parameters from the null space are the same as those initially found with the addition of the intercept and slope covariance along with the slope and quadratic covariance.

## 10 Discussion

In this article, we made several contributions to model identification, some of which were novel and some of which were not. We reviewed previously known results on model identification of parametric models of the multivariate Gaussian distribution, applying these results to SEMs with continuous variables, ordered categorical variables, constraints, and multiple groups. With modern computers, the method of local model identification is relatively simple. The method examines the mapping between the free parameters of the model and the model-implied summary statistics. If the first derivative of this mapping—called a Jacobian—has rank equal to the number of free parameters, then the model is locally identified. Because these results are not yet well-known in the SEM literature, communicating these results to the present audience might be the largest contribution of this work despite its lack of novelty. However, we paired this exposition with some extensions of the model identification method to two new situations. First, we extended local model identification to the case of very general exogenous covariates called definition variables which can modify any part of an SEM in extremely flexible ways. Second, we proposed an extension of standard local model identification to empirical identification by incorporating information on the patterns of missing data. To make these mathematical and theoretical contributions more concrete, we illustrated their application to several synthetic modeling tasks and to a real data analysis from the NLSY. Finally, we provide a software tool in the open-source OpenMx package in R that implements these solutions and makes them freely available to researchers in the mxCheckIdentification() function.

As with any method, the previously known and presently proposed methods for model identification have their shortcomings. The largest limitation is that all of the model identification checks discussed here—including those for definition variable and empirical identification—are strictly for *local* model identification, not global identification. A model can be locally identified for a particular set of parameter values, and yet have a non-unique set of optimal free parameters. Choosing appropriate latent variable scaling methods and setting plausible bounds on free parameters can limit the impact of multiple minima at the costs of requiring researcher foreknowledge of solutions and limiting potentially valid alternative solutions. Moreover, a model can be locally unidentified for one set of free parameters, and yet be locally identified for the vast majority of possible parameter values. Testing local identification under a variety of perhaps pseudo-randomly selected parameter values can overcome small regions of parameter space where local identification fails.

A further limitation of the present model identification approach may be its implementation in the OpenMx software. The flexibility of model specification in the OpenMx software mandates either extremely sophisticated algorithms for symbolic matrix calculus or reliance on numerical solutions for computing the Jacobian and its rank. In rare cases, the numerically determined rank of a matrix can differ from the analytic rank. Consequently, the computed identification of such a model could be inaccurate. In our experience, this is exceedingly rare and is often solved through recalculating identification after psuedo-random variation of the free parameters. Furthermore, for some researchers model specification in OpenMx can be challenging compared to other software. Fortunately, other packages exist which can ease this model specification. The EasyMx package (Hunter, 2022) offers wrapper functions for common modeling tasks, and the mxsem package (Orzek, 2023) offers a model-specification syntax based on that of the lavaan package (Rosseel, 2012).

In principle, the same method for identifying parametric models of the Gaussian distribution applies to mixed effects models as well. In this case, identification relies more heavily on the fixed effects design matrix and the random effects design matrix. There is also some degree of added complication about correctly choosing the summary statistics for mixed effects models. These must be defined at the cluster level. Moreover, generalized mixed effects models add some non-Gaussian difficulties to the identification approach undertaken here. Overall, the same mathematical theorems should apply to the case of mixed effects models, but it seems far from trivial to make this application.

We should also note that all the model identification techniques discussed here are for frequentist modeling only. Bayesian model identification requires an entirely different mathematical framework from that used here, one that obviates many issues in frequentist identification. In his classic monograph on Bayesian statistics, Lindley (1972, p. 46) offhandedly remarked that identification is rarely a problem for Bayesian models. Although rare, identification of Bayesian models remains a matter of concern. Palomo et al. (2007) presented an accessible introduction to Bayesian models and identification, and Florens & Simoni (2021) recently elucidated many more details specific to identification.

Limitations notwithstanding, local model identification can help researchers solve a variety of theoretical and empirical problems. From planning appropriate research designs to resolving non-convergent model estimation, model identification is a key preliminary step to almost all data analysis questions. Combining previously known results and extending them to new cases, we present a software implementation that checks for model identification in the OpenMx software. The software not only determines whether or not a given model is identified at user-provided or estimated parameter values, but also outputs which parameters are not identified, if any. The same tool can uncover issues related to empirical identification. With the availability of such a tool, model identification for SEMs need not remain shrouded in mystery.

## Data Availability

Code to run all analyses is publicly available as well as simulated data. Real data are maintained and secured by the National Longitudinal Survey of Youth.

## Footnotes

1 Note that the terminology commonly used is not particularly precise or formal. It would be more precise to refer to the “factor set of matrices,” “LISCOMP set of matrices,” and the “RAM set of matrices” rather than the “factor model,” “LISCOMP model,” and the “RAM,” but the gain in precision leads to exceedingly uncommon phrasing.

2 Bekker & Wansbeek (2001, pp. 151–153) calls these constraints “prior information.”

3 If we add the requirement that each underlying latent variable is independent conditional on the conventional SEM latent variables, then we are creating a probit model for the ordinal variables. However, this conditional independence is not a requirement for identification: the underlying latent continuous variables can be mutually correlated. As Skrondal & Rabe-Hesketh (2004, Chapter 5) noted, univariate probit and logit models for the underlying latent variables have different identification requirements.

4 Alternatively and equivalently, the observed proportion of each response category can be added to the observed statistics (see Hunter et al., 2023, p. 57, Equation (52) for the relevant equivalence in the Jacobian)

5 To allow different variables to have a differing number of thresholds, this matrix may be jagged.

6 This “multilevel” use of definition variables is analogous to dummy-coding an ID variable with *k* levels into  variables and adding them all as fixed effects. In contrast, true multilevel modeling uses the ID variable to create a random intercept. In this sense, models with definition variables are certainly *not* multilevel models.

## A Technical and formal details of model identification

Let  be a parametric model of the multivariate random variable  with free parameter vector . Furthermore, suppose that *Y* follows a known probability function  which varies based on , and that  represents some observed value from the random variable . Finally, suppose all possible free parameters are elements of a set  , which is an open subset of  when there are *J* free parameters. The definitions below closely follow those of Bekker & Wansbeek (2001) and Magnus and Neudecker (1988, p. 333).

Two parameter points  and  are called *observationally equivalent* if  for all possible . The *k*th element of the free parameter vector  is notated  . The element  is called *locally identified* if there exists an open neighborhood of  such that no point  is observationally equivalent to  with  . Stated less formally, an element of the free parameter vector is locally identified at some particular value when you can find a region of parameter space in which there are no observationally equivalent points for that element other than that element itself. The vector of free parameters is locally identified when all of the elements are locally identified. An element of the free parameter vector is called *globally identified* when the open neighborhood of its local identification is the entire parameter space  , and similarly for the free parameter vector being globally identified (Bekker & Wansbeek, 2001, p. 147, Definition 4). An immediate consequence of a locally identified model is that there is only one vector of optimal parameter values in the neighborhood of . Similarly, an immediate consequence of global identification is that there is only one globally optimal vector of parameters throughout parameter space.

In the case of a continuous, multivariate Gaussian random variable , the probability function  is the multivariate Gaussian probability density function. A multivariate Gaussian distribution is completely specified by its mean vector  and covariance matrix , both of which we assume are functions of the free parameter vector . The model  of a Gaussian random variable can then be thought of as a mapping from the free parameter vector to the mean vector  and covariance matrix . Mathematically, this mapping is shown in Equation (1). Every model  of a Gaussian random variable has its own corresponding mapping .

Two parameter points  and  of a Gaussian model will be observationally equivalent whenever they imply the same mean vector and covariance matrix. Stated mathematically, two parameter points of a Gaussian model are observationally equivalent if . A Gaussian model is locally identified at some parameter value  if there are no observationally equivalent points in the neighborhood of . Global identification of a Gaussian model is then defined by extending the neighborhood of local identification across the entire parameter space  .

Starting with the definition for observational equivalence, all the definitions provided above depend on all possible values of . However, in practice with real data we have never observed all possible values of ; rather, we observe some finite set of values of . Thus, observational equivalence, local identification, and global identification are all mathematical and theoretical concepts that are independent of the actual data we observe. Empirical identification particularizes these theoretical concepts to an actual observed set of data. Therefore, two parameter points  and  are called *empirically observationally equivalent* if  for all observed values of . This definition is almost identical to that for observational equivalence; the exception is that instead of equality over all possible values of , we are restricted to a subset of these possible values that were actually observed. Parallel definitions for empirical local identification and empirical global identification follow straightforwardly from empirical observational equivalence.

The utility of these definitions is often in their negative cases. We need to find points that are *not* observationally equivalent. For example, suppose two points are *not* observationally equivalent for all possible , but they are obervationally equivalent over the particular observed values of . This situation would lead to a model that is locally identified, but not empirically identified.

In common shorthand, we refer to locally identified models as identified models, and append the modifier “global” when necessary. Similarly, we speak of empirically identified models, but more formally mean locally empirically identified models.

## B Identification of ordinary least squares (OLS) regression and definition variables

Consider identification for OLS regression. The OLS regression model can be written as in Equation (B.1)

(B.1)

where  is an  vector of univariate outcomes,  is an  design matrix with one predictor variable in each column which may include a column of 1s for an intercept term,  is the vector of residuals with mean zero and variance  , and  is an  identity matrix. Note that  denotes a multivariate normal distribution with mean in the first slot and variance in the second. So, OLS regression can be considered a multivariate model of a Gaussian distribution.

The OLS regression model is known to be identified if and only if the predictors are linearly independent (i.e., not perfectly collinear predictors Greene, 2003, p. 13). Put another way, OLS regression is identified when the design matrix  is of full column rank. In what follows, we will first show how this identification result derives from the rank Jacobian criterion we discussed previously, and then we will show how the regression model can illustrate model identification for SEMs with definition variables.

To derive the identification criterion for OLS regression, we consider Equation (B.1) as a parametric model for the multivariate Gaussian distribution, and apply the same method of identification we have made previously. The vector of free parameters for Equation (B.1) is

(B.2)

The mapping from the free parameters to the observed distribution (i.e., summary statistics) is then

(B.3)

Observe that Equation (B.3) implies a potentially different mean for each of the *N* variates in the multivariate Gaussian distribution and a structured covariance matrix across variates. These *N* variates are the rows of data in OLS regression, so the model implies a potentially different mean for each row of data and independent covariances across rows. The Jacobian of  is given by

(B.4)

One can see that the Jacobian no longer depends on any free parameters. This lack of dependence on free parameters implies that the *local* identification for OLS regression also yields *global* identification (Bekker & Wansbeek, 2001, p. 155, Theorem 7). One can also see that the Jacobian is block-diagonal. Due to theorems on the rank of block-diagonal matrices, the rank of the Jacobian in Equation (B.4) will be the sum of the ranks of the blocks (see Roman, 2005, p. 46, Theorem 1.14 on the dimension of a direct sum of vector spaces and then invoke the isomorphism between direct sums of vector spaces and block-diagonal matrices). Moreover, the single column in  always necessarily has rank one. Therefore, the residual variance  is always identified in the sense that  will never be part of the null space that signifies non-identified parameters. The regression parameters in  are identified if and only if the design matrix  has full column rank. Put another way, the OLS regression model is identified if and only if the predictors are linearly independent (i.e., there are no perfect collinearities among the predictors).

Next we represent OLS regression as an SEM with definition variables to illustrate identification of SEMs with definition variables. As an SEM, OLS regression is a univariate Gaussian model of independent rows with the same variance for all rows, but with the mean being a function of a set of definition variables as given by Equation (B.5)

(B.5)

where  is the univariate outcome for row *i*,  is the *i*th row of the design matrix, and the remaining parameters are the same as described in Equation (B.1). Note that  can be alternatively conceived as a row vector of the definition variable values at data row *i*. The free parameters in Equation (B.5) are the same as in Equation (B.1) and are given by Equation (B.2). The summary statistics for the SEM in Equation (B.5) are the mean and variance of  which may be functions of definition variables. The summary statistics are then repeated for each row of data. Thus, the analog of Equation (B.3) is

(B.6)

where  is a vector of *N* 1s. Thus,  maps the  free parameters to  summary statistics: *N* means and *N* variances. The Jacobian of Equation (B.6) is then

(B.7)

The structure of the Jacobian in Equation (B.4) is highly similar to that in Equation (B.7). The primary difference is in how the variance is handled. In the multivariate model of Equation (B.1), we specified the full covariance matrix of  and therefore used the half-vectorization in Equations (B.3) and (B.4). By contrast, in the univariate SEM of Equation (B.5), we assumed rows were independent but were potentially functions of the definition variables. So, the univariate variance is repeated *N* times in Equation (B.6). When differentiated, the repeated variance becomes a column of 1s in Equation (B.7) instead of the half-vectorization of the identity matrix as in Equation (B.4).

The rank of the Jacobian in Equation (B.7) is determined quite similarly to that in Equation (B.4). The residual variance  is always identified (i.e., it can never be part of the null space that indicates non-identified parameters), and the free parameters  are identified if and only if the design matrix  is of full column rank (i.e., linearly independent predictors, also known as not perfectly collinear predictors). Thus, we have shown that expressing OLS regression as an SEM with definition variables yields the exact same identification criterion as typical identification of OLS regression.

Beyond the case of OLS regression, identification of general SEMs with definition variables merely expresses the conventional Jacobian for each row of data. This process creates a multigroup model with one group for each row of data, and identifies the multigroup model. More precisely, it creates one group for each unique combination of definition variable values. In the most general case of *N* unique rows of data with *p* definition variables, *v* modeled variables, and *k* free parameters, the Jacobian has  rows and *k* columns. That is, there are *N* rows in the Jacobian for each summary statistic (mean, variance, and covariance) and one column for each free parameter.

Although the full Jacobian with  rows is technically required for identification, in practice only a small subset of *N* is often sufficient. Many models with definition variables are identified with one or two unique combinations of definition variable values. If two unique combinations of definition variable values is sufficient, then there are only two rows in the Jacobian for each summary statistic (mean, variance, and covariance) and one column for each free parameter, thus keeping the Jacobian relatively small and computationally tractable.
